# High wear resistance of femoral components coated with titanium nitride: a retrieval analysis

**DOI:** 10.1007/s00167-017-4578-7

**Published:** 2017-05-20

**Authors:** Christian Fabry, Carmen Zietz, Axel Baumann, Reinhard Ehall, Rainer Bader

**Affiliations:** 1 Biomechanics and Implant Technology Research Laboratory, Department of Orthopaedics, University Medicine Rostock, Doberaner Str. 142, 18057 Rostock, Germany; 2 DOT GmbH, Charles-Darwin-Ring 1a, 18059 Rostock, Germany; 3 Graz Ragnitz Private Hospital, Berthold-Linder-Weg 15, 8047 Graz, Austria

**Keywords:** Polyethylene, Retrieval analysis, Surface damage, Failure analysis, Ceramized surface, Wear, TiN, Coating, Total knee arthroplasty

## Abstract

**Purpose:**

The objective of this study was to evaluate the in vivo wear resistance of cobalt-chromium femoral components coated with titanium nitride (TiN). Our null hypothesis was that the surface damage and the thickness of the TiN coating do not correlate with the time in vivo.

**Methods:**

Twenty-five TiN-coated bicondylar femoral retrievals with a mean implantation period of 30.7 ± 11.7 months were subjected to an objective surface damage analysis with a semi-quantitative assessment method. A visual examination of scratches, indentations, notches and coating breakthroughs of the surfaces was performed. The roughness and the coating thickness of the TiN coating were evaluated in the main articulation regions.

**Results:**

Narrow scratches and indentations in the range of low flexion angles on the retrieval surfaces were the most common modes of damage. There was no evidence of delamination on the articulation surface but rather at the bottom of isolated severe indentations or notches. An analysis of three retrievals revealed a coating breakthrough in the patellofemoral joint region, resulting from patella maltracking and a dislocation. The arithmetical mean roughness of the TiN surface slightly increased with the implantation period. In contrast, the maximum peak height of the roughness profile was reduced at the condyles of the retrieved components in comparison with new, unused surfaces. No significant association between the coating thickness and implantation period was determined. Moreover, the measured values were retained in the range of the initial coating thickness even after several years of in vivo service.

**Conclusions:**

As was demonstrated by the results of this study, the surface damage to the TiN coating did not deteriorate with the implantation period. The calculated damage scores and the measured coating thickness in particular both confirmed that the TiN coating provides low wear rates. Our findings support the use of wear-resistant TiN-coated components in total knee arthroplasty with the objective of reducing the risk of aseptic loosening. However, in terms of TiN-coated femoral components, particular attention should be paid to a correct patellar tracking in order to avoid wear propagation at the implant.

## Introduction

The most common bearing couple in total knee arthroplasty (TKA) comprises a femoral component and tibial tray that are made of titanium or cobalt-chromium (CoCr)-based alloys, respectively, and are combined with an ultrahigh molecular weight polyethylene (UHMWPE) insert. Wear has been implicated as one of the major factors affecting the long-term clinical performance of these components, which can be mainly attributed to: adhesion, abrasion and fatigue mechanisms during the sophisticated knee kinematic of rolling and sliding [10]. In vitro simulator studies have shown that roughened counterfaces increase the UHMWPE wear [5], caused by undesired conditions, such as third-body wear, or simply by the cyclic joint motion over the years in vivo [30].

Depositing a ceramic coating like titanium nitride (TiN) on the surface in a range of a few microns is an alternative method of increasing the abrasion resistance of metal components made of cobalt-chromium- or titanium-based alloys. This biologically inert surface modification leads to an increase in surface hardness, while maintaining the strength and toughness of the metal substrate [28]. Further, the TiN coating acts as a barrier that inhibits the release of ions from the metal substrate, protecting the patient from allergy-inducing ions like chromium or cobalt [26, 27].

Whereas TiN coatings have a long history in TKA, the post-operative clinical outcome for those coated implants has been underrepresented in arthroplasty literature. In the few clinical studies concerning the implant survival of CoCrMo TKAs with TiN coatings, revision rates of <5% have been reported with a mean follow-up of 79 and 60 months, respectively [19, 29]. Differences in clinical and functional parameters were not found in patients without known hypersensitivities against implant materials in coated or uncoated TKAs after a follow-up period of 5 years [29]. Except for the studies in which revision rates were documented, the damage to TiN-coated TKAs and the reasons for revision were previously unknown.

Retrieval analyses have received more attention in the last years as these studies have provided an exclusive insight into how wear affects the hard femoral component besides the more softer insert made of different types of polyethylene [2, 14, 17, 24]. Currently, no data exist to explain the sensitivity of TiN-coated TKAs made of CoCrMo with regard to surface damage and wear resistance. Therefore, the aim of this study was to asses the surface damage of TiN-coated femoral components retrieved from patients who underwent total knee revision surgery using a visual semi-quantitative assessment method. The clinical relevance of different coating damage modes was also discussed. In addition, detailed surface roughness and coating thickness measurements were taken in the most highly strained regions of articulation for the first time in order to analyse the proneness of the coated surfaces to third-body wear.

Taking the retrieved femoral components into consideration, we proposed the hypothesis that the surface damage and the thickness of the TiN coating do not deteriorate with the implantation period.

## Materials and methods

### Demographics

Approval was granted to enable the analysis of 25 femoral components, retrieved from 25 consecutive total knee revision surgeries that were performed at the Graz Ragnitz Private Hospital between 2011 and 2015. The duration of implantation ranged from 4.9 to 94.1 months. At the time of revision, the mean patient age was 67 years (range 53–82). The mean body mass index (BMI) was 30.0 kg/m^2^ (range 22.0–44.9). Infection and septic loosening were the principal reasons for revision. Other reasons as well as all the relevant patient data are summarized in Table 1.

**Table 1:** Patient data for the retrieved total knee implants (mean ±95% confidence intervals)

Variables	
Patients	25
Males	15
Females	10
Side of implantation	
Right	14
Left	11
Age (years)	67.7 ± 3.2
Mass (kg)	86.6 ± 8.5
Height (cm)	169.3 ± 3.2
BMI (kg/m^2^)	30.0 ± 2.5
Duration in situ (months)	30.7 ± 11.7
Year of coating	
Coated before 2012	12
Coated as from 2012	13
Reasons for revision	
Infection	11
Septic loosening	4
Ligament instability	2
Pain	2
Aseptic loosening	1
Patella dislocation	1
Tibia dislocation	1
Inlay dislocation	1
Fracture	1
Recurrent effusion	1

The examined femoral components were all of a bicondylar, TiN-coated mobile-bearing TKA design (ACS^®^ MB system, Implantcast, Buxtehude, Germany) of different sizes. Prior to the analysis, all components were sterilized and carefully cleaned with acetone in accordance with former retrieval studies [14, 15, 24]. Every component was then photographed with a digital camera (LUMIX DMC-FZ28, Panasonic, Kadoma, Japan).

### Damage assessment

**Fig. 1:**
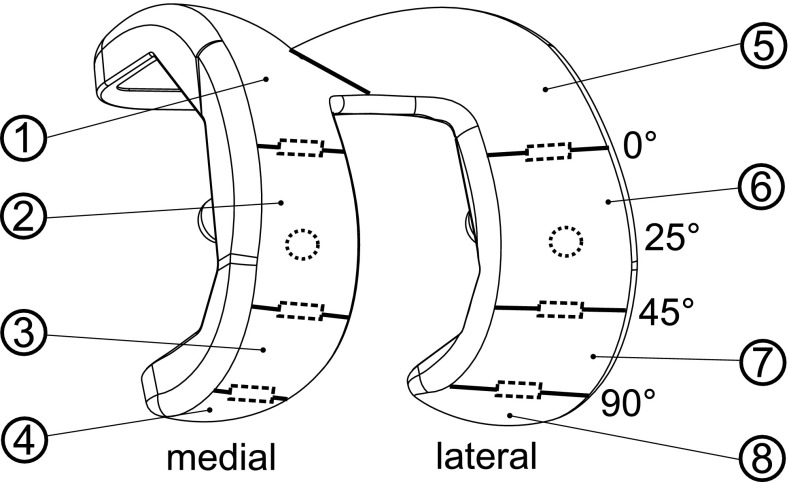
Shematic illustration of the defined sections (*1*–*8*). The *dashed rectangles* represent the sections for roughness measurements at 0°, 45° and 90° of flexion. In addition, the locations for the coating thickness analyses at 25° of flexion angle are highlighted with the *dashed circles*

The surface damage was analysed on the femoral components by using the modified semi-quantitative grading method presented by Brandt et al. [3]. The components were subdivided into eight defined sections (Fig. 1) for this purpose.

Two observers (CF and CZ) independently evaluated the regions with the naked eye, as well as with the help of a stereo-light microscope (Lynx EVO, Vision Engineering Ltd., UK) at a magnification of 40. Localized damage was identified (Table 2).

**Table 2:** Summary of the specific damage modes used in the analysis

Damage mode	Definition
Scratches	Minimum of 2 mm in length and primarily formed in the main direction of stress (anterior–posterior)
Coating breakthrough	Destruction of the coating layer caused by wear
Indentations	Small pits
Notches	Kind of plastic deformation caused by an impact

Damage that was caused by the revision procedure, which was evidenced by untypical cross-passing scratches or instrument imprints, was not examined within this analysis. In order to illustrate the characteristics of the damage, high-resolution images with magnifications up to 400× were taken with the help of a laser scanning microscope (VK-X260, Keyence, Osaka, Japan).

Damage evaluation was performed with the damage feature score (DFS), comprising the product of an area score and a severity score. The area score included a classification from 0, 1, 2,…10, which corresponded to an estimated damage region (DA) of DA = 0%, 0% < DA ≤ 10%, 10% < DA ≤ 20% and … 90 < DA ≤ 100% [3].

In addition, the severity score was classified into four categories and was dependent on the mode of damage (Table 3).

**Table 3:** Classification of the severity score

Scratches	
0	No damage
0.33	Visible with intact coating
0.66	Visible with coating breakthrough, but no damage to the substrate
1	Coating breakthrough in combination with grooving at the substrate
Coating breakthrough	
0	No damage
0.33	Rare, sporadic
0.66	Extensive, sharp transition to the coated area
1	Extensive with frayed transitions to the coated area, attributed to third-body wear
Indentations	
0	No damage
0.33	Mild with intact coating
0.66	Coating is partly removed in the crater or in the edge region
1	Coating is fully removed from the indentation
Notches	
0	No damage
0.33	Mild with intact coating
0.66	Coating is partly removed in the crater or in the edge region
1	Coating is fully removed from the indentation

Thus, eight sections per femoral component were evaluated with the help of the DFS. The sum of the corresponding region-related DFSs generated the total femoral damage score (FDS); see Eq. (1).

1 $${\text{FDS}} = \sum\limits_{i = 1}^{n} {{\text{DFS}}_{i} }$$

### Profilometry

The surfaces of all femoral components were measured at three defined sections: 0°, 45° and 90° of flexion on each condyle (Fig. 1), as suggested by Brandt et al. [3]. A contact profilometer (Hommel Tester T8000 Wave, Jenoptik, Wedel, Germany) was used to determine: the arithmetical mean surface roughness (*R*_a_), the maximum profile peak height of the roughness profile (*R*_p_), the total height of the roughness profile (*R*_t_) and the root-mean-square deviation of the roughness profile (*R*_q_). The parameters during the measurements included a stylus tip radius of 2 µm, a traversed length of 1 mm, as well as a measurement speed of 0.15 mm/s^−1^. These parameters ensured an accuracy of 0.01 µm which was verified with a reference standard before measurement.

Three roughness measurements were taken on the aforementioned sections (0°, 45° and 90° of flexion), producing 18 measurements per femoral component. In addition to the retrievals, three new, unused TiN-coated femoral components of the same design (ACS^®^ MB femoral component, Implantcast GmbH, Buxtehude, Germany) were measured on the same sections to provide an objective evaluation of the roughness parameters.

### Determination of coating thickness

The coating thickness was measured by using the crater grinding method in accordance with the standard EN 1071-2 [7] for curved surfaces. The measurements were taken at 25° flexion on both the medial and lateral condyles (Fig. 1). A combined motion of rollback and sliding occurs in this area of contact during walking [1], representing the most frequent and wear-intensive activity in daily living [23]. During the transition from the rolling to the sliding motion, the shear stress between the articulating components reaches its maximum in the anterior–posterior direction [8]. Thus, the highest amount of frictional load acting on the coating during movement can be expected in this region.

A spherical abrasion testing device (kaloMAX NT II, BAQ GmbH, Braunschweig, Germany) was used to generate the penetrations. A 20-mm-diameter stainless steel ball was rotated with a velocity of 500 rpm onto the coated femoral components for 30 s, grinding a spherical cavity, supported by a droplet of diamond suspension (Calotest-hq, Eifeler Suedcoating GmbH, Ettlingen, Germany). Afterwards, each crater was measured from above with an accuracy of 0.1 µm (Software ProfCoat 1.0, MS-International, Rostock, Germany), and the inner and outer radii of the ring that separates the worn coating region from the intact coating section were determined.

For an objective evaluation of the correlation between coating thickness and in vivo duration, the retrievals were grouped with regard to the year of coating (first group: before 2012 with *n* = 12, and second group: coated as from 2012 with *n* = 13). This classification was based on the fact that starting from 2012, the initial coating thickness was raised by the manufacturer from 4.5 ± 1.5 to 5.5 ± 1.5 µm. A clear allocation was possible with the help of the LOT and REF numbers which provided the information about the year of coating.

### Statistical analyses

The presented data are shown as a mean value ±95% confidence interval. The statistical significance of the differences of the damage feature score (DFS) and the femoral damage score (FDS) between the observers was assessed using the independent Mann–Whitney *U* test [IBM^®^ SPSS^®^ Statistics Version 20 (IBM Corporation, New York, USA)]. *P* values of <0.05 were considered significant. The correlation of different parameters to the implantation period was determined in accordance with Pearson. The value of the correlation coefficient was used to interpret the form of correlation in the following steps: 0 < *r* ≤ 0.2 means very low correlation; 0.2 < *r* ≤ 0.5 means low correlation; 0.5 < *r* ≤ 0.7 means average correlation; 0.7 < *r* ≤ 0.9 means high correlation; and 0.9 < *r* ≤ 1 represents a very high correlation.

Due to the lack of TiN-coated femoral components of identical implant design in the retrieval archives, the present study was based on a limited number of samples. As all of these available retrievals were used in the analysis, a power calculation was not performed.

## Results

### Damage assessment

**Fig. 2:**
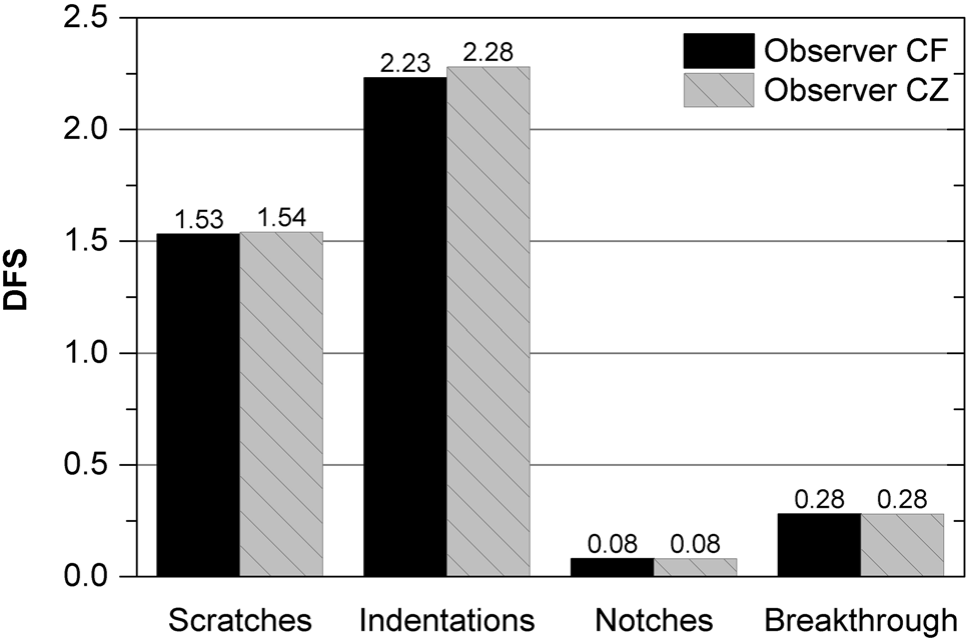
Damage feature scores with regard to all retrieved femoral components (mean) obtained by the observer CF and CZ for the analysed damage modes

In general, minor surface damage on the femoral components was evidenced by the DFS (Fig. 2). The scratch DFS was the highest followed by the indentation DFS. Sparse directional scratches were detected in the main contact regions of the 25 femoral condyles (sections 2, 3, 6, 7). Only with three components, the coating was worn through at the bottom of individual scratches in combination with grooving at the substrate. The scratches on all other retrievals did not penetrate the coating.

**Fig. 3:**
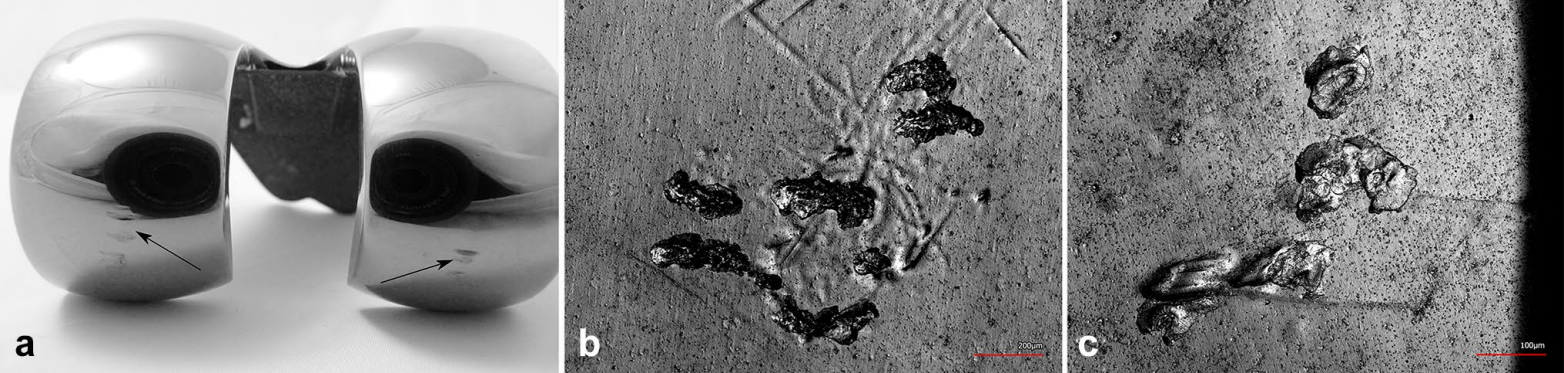
Damage modes: **a** big notches at the posterior condyles, *highlighted* by *arrows*, **b** typical indentations in the damage regions 2 and 6 at a magnification of 200x without delaminations, **c** signs of delaminations in the bottom of indentations at a magnification of 400x

Indentations were mostly located in the range of 0° of flexion (sections 2, 6) and were approximately 200 µm in size (Fig. 3b). Small areas suggesting delamination were observed at the bottom of the indentations (Fig. 3c) of two femoral components.

An extremely low number of notches in the posterior condyle region (sections 4, 8; Fig. 3a) were detected, which was evidenced by the very low DFS (Fig. 2). One of two components with notches exhibited a delamination at the bottom of the notch.

**Fig. 4:**
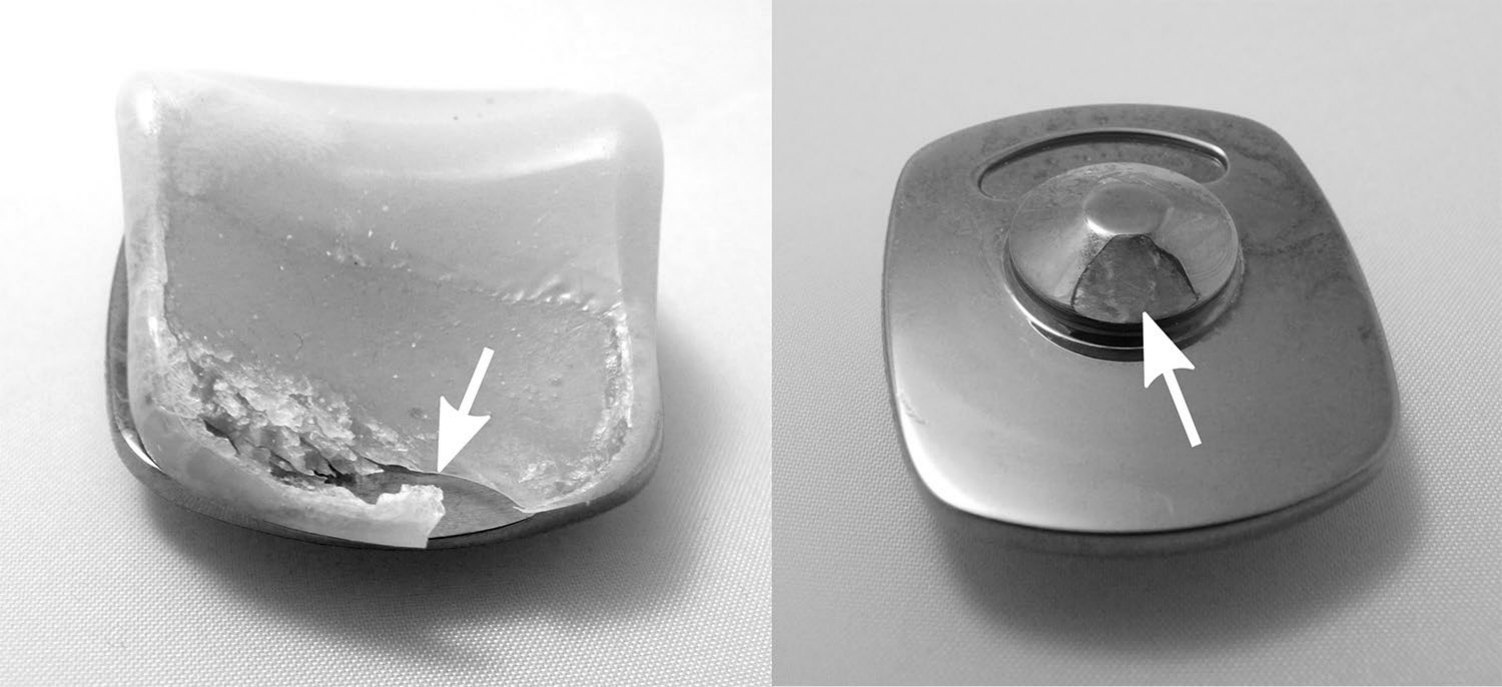
*Left* unevenly worn metal base plate of the patella resurfacing; *right* worn metal base plate with coating breakthrough as a result of a patella dislocation

**Fig. 5:**
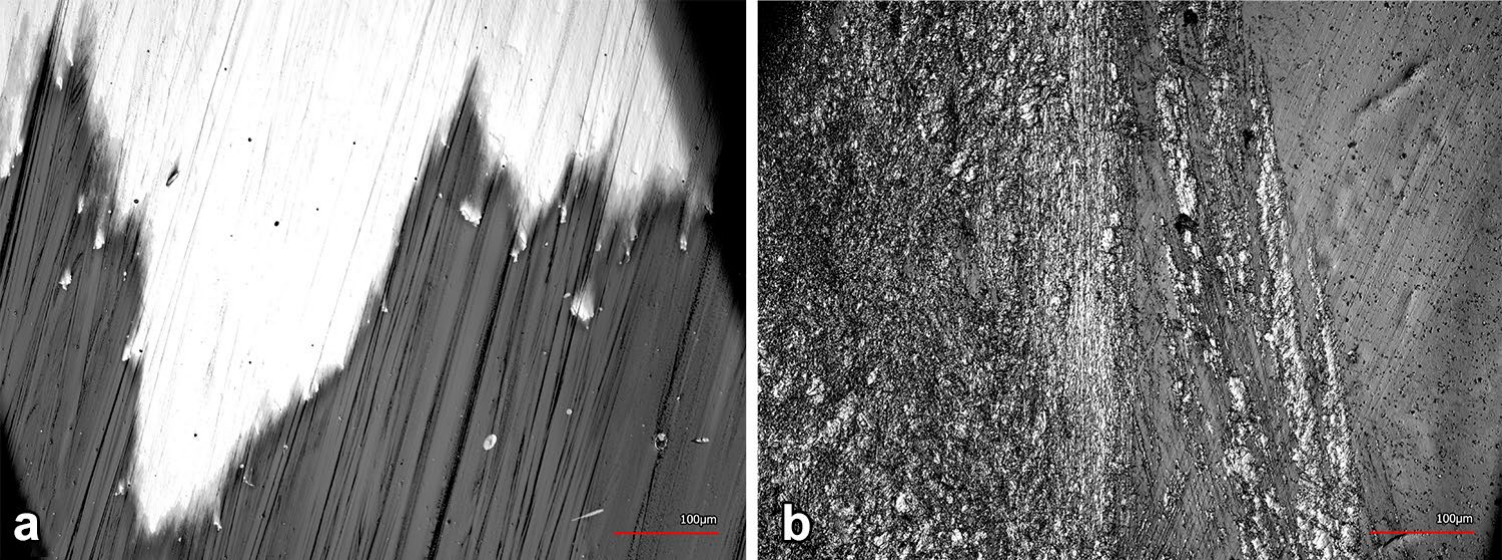
Edge regions of coating breakthrough (lighter regions = substrate material, darker regions = TiN coating) at a magnification of 400x: **a** coating breakthrough in combination with the native patella, **b** coating breakthrough resulting from a metal-on-metal contact

A coating breakthrough was found in the patellofemoral joint region (sections 1, 5) of three femoral components which were revised after a respective implantation period of 10.2, 15.6 and 64.4 months, respectively. The estimated damaged area was in the range of 10–20% of the respective section. In two cases, the analysis of the corresponding patella resurfacing evidenced the direct contact between the metallic base plate and the femoral component (Fig. 4). In the third case, no patella resurfacing was implanted in combination with the examined TiN-coated mobile-bearing TKA. However, the visual appearance of the coating breakthrough differed from the first two cases. Here, the edges of the narrow coating breakthrough were obvious and smooth, whereas the edge regions of the coating failure, which resulted from metal-on-metal contact, were irregular and rough (Fig. 5).

**Fig. 6:**
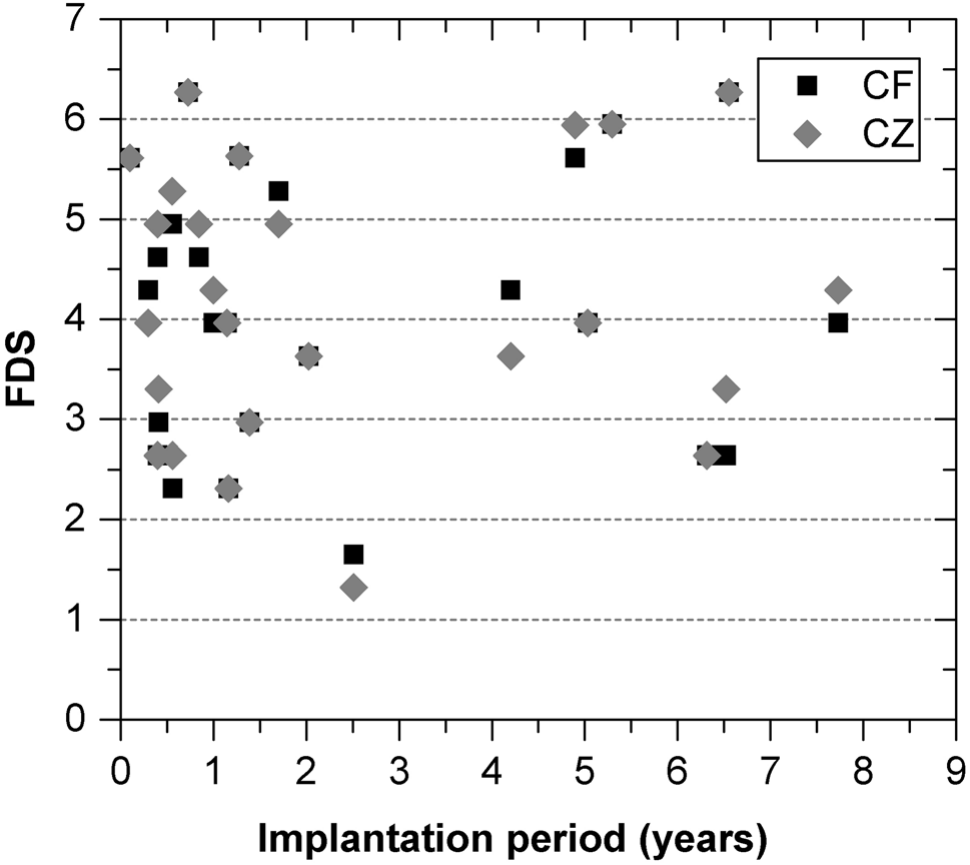
Calculated femoral damage score (FDS) obtained by the observers CF and CZ with implantation period for all retrievals

None of the calculated FDSs for all retrievals correlated with the implantation period (*r* = 0.061) (Fig. 6). The mean values for the FDS were 4.12 (observer CF) and 4.19 (observer CZ), respectively. Both the DFS and the FDS showed no significant differences between the observers.

### Profilometry

Surface roughness parameters were determined at different sections on the retrieved femoral components when comparing them with those measured at new, unused femoral components (Table 4). The arithmetical mean surface roughness *R*_a_ for the retrieval group was slightly higher than the values of the unused control group, with the exception of 90° of flexion (medial). As expected, the same trend with the exception of 90° of flexion (medial) was applicable to the root-mean-square deviation of the roughness profile *R*_q_. The total height of the roughness profile *R*_t_ was also increased for the retrieved condyles (Table 4). The 90° section of medial flexion showed a deviation from all other sections as well.

**Table 4:** Surface parameters of the femoral condyles at different sections (mean ±95% confidence interval)

	Medial femoral condyle			Lateral femoral condyle		
	0°	45°	90°	0°	45°	90°
*R*_a_ (µm)						
Retrieval	0.03 ± 0.01	0.03 ± 0.00	0.02 ± 0.01	0.03 ± 0.00	0.03 ± 0.01	0.03 ± 0.01
Controls	0.02 ± 0.01	0.02 ± 0.00	0.03 ± 0.01	0.02 ± 0.00	0.02 ± 0.01	0.02 ± 0.01
*R*_q_ (µm)						
Retrieval	0.05 ± 0.06	0.05 ± 0.04	0.04 ± 0.01	0.04 ± 0.01	0.04 ± 0.01	0.05 ± 0.01
Controls	0.04 ± 0.01	0.04 ± 0.01	0.05 ± 0.01	0.03 ± 0.01	0.04 ± 0.01	0.04 ± 0.02
*R*_p_ (µm)						
Retrieval	0.22 ± 0.05	0.26 ± 0.07	0.22 ± 0.05	0.21 ± 0.03	0.22 ± 0.06	0.28 ± 0.05
Controls	0.32 ± 0.08	0.39 ± 0.08	0.5 ± 0.04	0.17 ± 0.06	0.34 ± 0.08	0.39 ± 0.21
*R*_t_ (µm)						
Retrieval	0.57 ± 0.12	0.58 ± 0.10	0.50 ± 0.10	0.54 ± 0.12	0.59 ± 0.13	0.60 ± 0.11
Controls	0.54 ± 0.07	0.48 ± 0.07	0.7 ± 0.11	0.33 ± 0.09	0.43 ± 0.12	0.57 ± 0.21

**Fig. 7:**
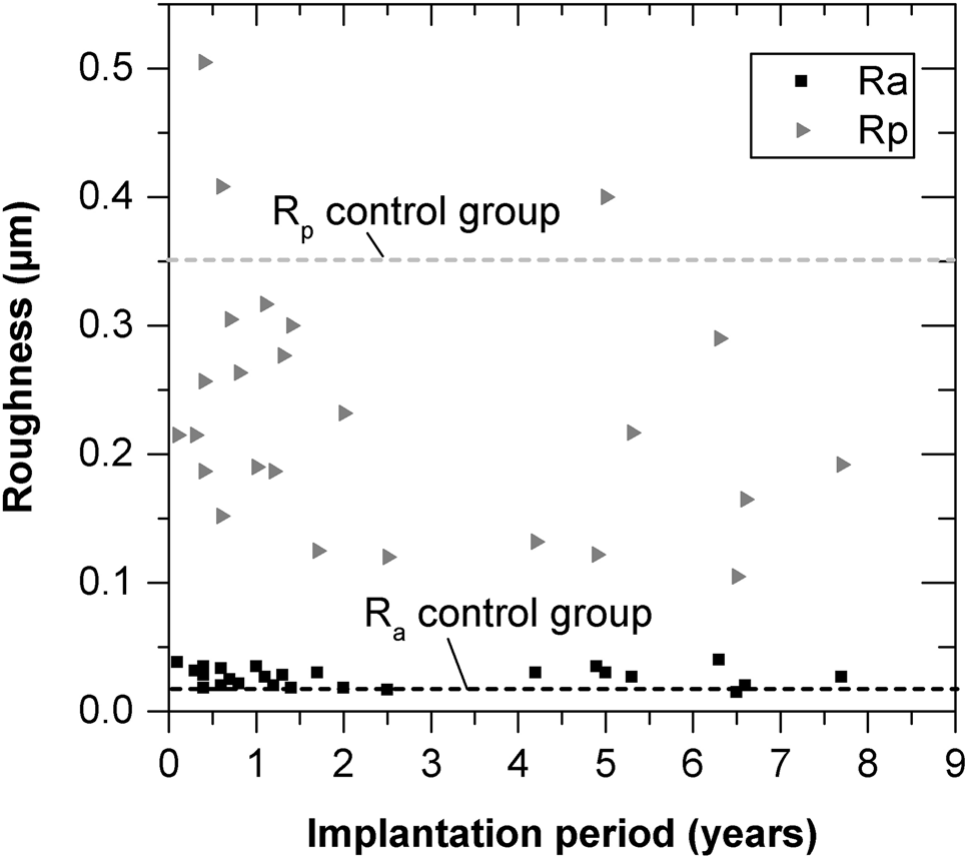
Overall roughness parameters *R*_a_ and *R*_p_ with time period after implantation for all 25 retrievals

In contrast, the maximum profile peak height *R*_p_ was mainly reduced at the condyles of the retrieved components, compared with the new, unused components. The correlation between the arithmetical mean surface roughness *R*_a_ and the implantation period, was very low (*r* = −0.037). Additionally, the correlation between the maximum profile peak height of the roughness profile *R*_p_ and the implantation period, was also low (*r* = −0.288). Therefore, a direct correlation between surface roughness and implantation time could be excluded (Fig. 7).

In general, the surface parameters were found to be higher on the medial condyles of the retrievals than on the lateral condyles. The only exception was the 90° section, where the roughness parameters were lower than the lateral side on average. However, this was also found in the control group with the unused components.

### Coating thickness

**Fig. 8:**
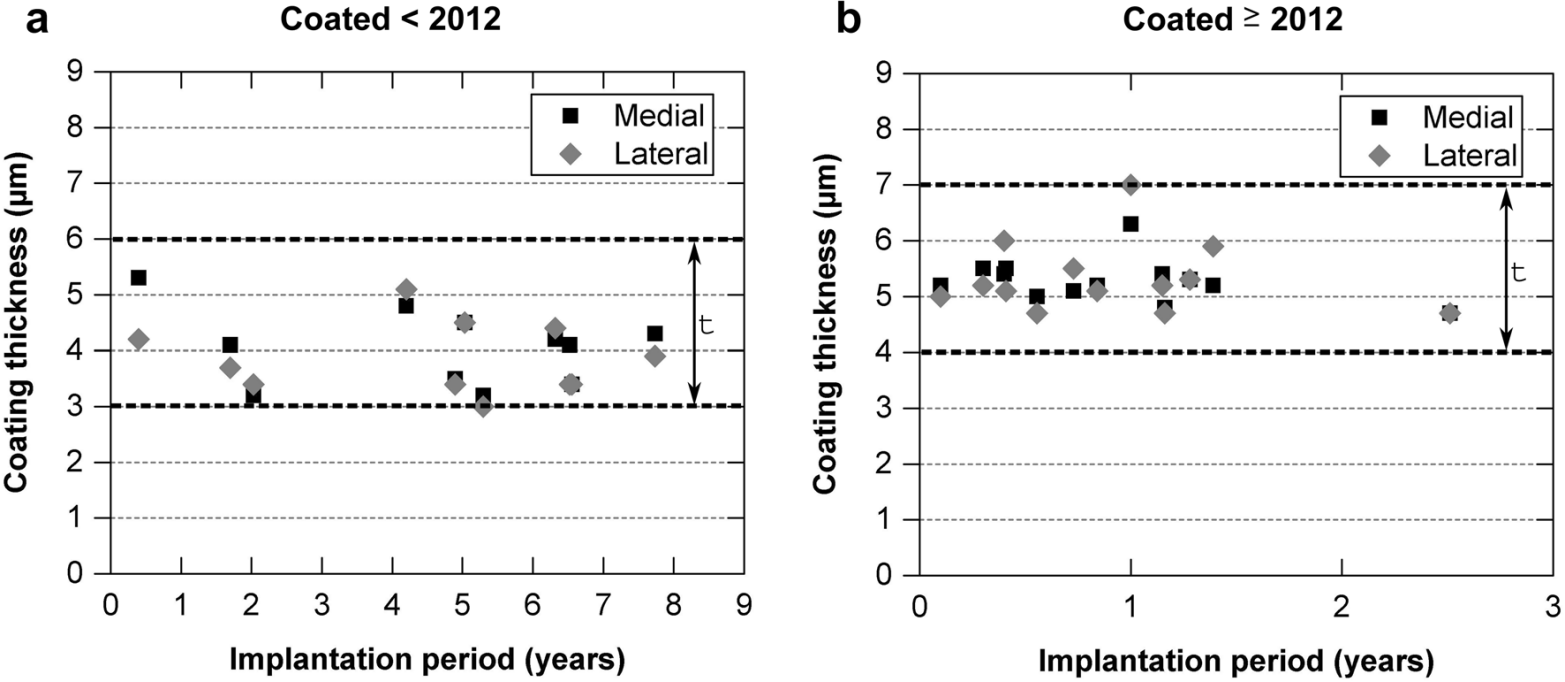
Coating thickness with implantation period for **a** retrievals coated before 2012 and **b** retrievals coated as from 2012. The parameter *t* represents the initial coating thickness range

The mean coating thickness was 4.0 ± 0.4 µm (mean ±95% CI) at the medial condyle and 3.9 ± 0.3 µm (mean ±95% CI) at the lateral condyle (Fig. 8a) for the retrievals coated before 2012. In contrast, the mean coating thickness for the retrievals coated from 2012 was increased with 5.3 ± 0.2 µm (mean ±95% CI) at the medial condyle and 5.3 ± 0.4 µm (mean ±95% CI) at the lateral condyle (Fig. 8b). A correlation between coating thickness coated before and after 2012 and the implantation period was not found (<2012: medial: *r* = −0.117, lateral: *r* = −0.076; >2012: medial: *r* = −0.357, lateral: *r* = −0.094).

## Discussion

### Damage assessment

The results of the visual semi-quantitative assessment method have shown that the surface damage of the TiN coating did not correlate with the implantation period. Four different types of damage were detected on the coated articulation surfaces, whereby narrow parallel scratches, similar to former retrieval studies, were primarily observed [13, 14, 17]. Scratches occur when scattered hard particles interact between the counterfaces within the sliding gap and abrade the coating [10]. The scratches that were evaluated were formed in the main direction of sliding (anterior–posterior) and differed in their orientation from the untypical cross-passing ones resulting from implantation or revision procedure. A coating breakthrough was detected at the bottom of separated narrow scratches in three retrievals. Severe third-body wear was responsible for the occurence of this coating failure. Due to its small dimension, the influence on the ion release behaviour may be extremely low. However, the recorded damage feature score for scratches was ten times lower than the one reported in a previous study [3] with femoral knee components made from CoCr and nearly three times lower than the score for retrieved, oxidized zirconium (OxZr) TKAs, despite them being implanted for a longer period (TiN: 30.7 months, CoCr: 23.1 months, OxZr: 21.9 months). TiN coatings are therefore assumed to be more resistant against third-body particles compared to CoCr or OxZr. In view of the clinical relevance of third-body particles [6], this could be an advantage for TiN-coated TKAs.

Besides scratches, indentations were the type of damage detected most frequently. In comparison with uncoated TKAs made of CoCr or OxZr, the indentation score was nearly 50% lower with the TiN coating [3]. Indentations were primarily located in the range of low flexion angles. The left pits may indicate a plastic deformation of the coated substrate, caused by third bodies during the stance phase. Notches were hardly observed and may be attributed to a heavy impact between the femoral component and the tibial tray, during movement at high flexion angles, as already mentioned by [4]. Generally, the TiN-coated retrievals revealed a notch score two times lower than uncoated CoCr and OxZr [3].

In two retrievals however, the TiN coating was partly delaminated on the bottom of an indentation and also at the bottom of a notch in one retrieval. These signs of delamination developed as a result of a massive plastic substrate deformation which was not fully covered by the elasticity of the TiN coating. However, this kind of delamination differed significantly from former findings, where wide regions of flaked coating fragments were detected as a result of insufficient adhesion at the substrate/coating interface [22]. Further, the sparse delaminations evaluated in this study were not located directly at the articulating surface but rather inside a pit and, thus, had no direct contact to the counterface. Therefore, surface asperities caused by delamination may not be associated with adhesive wear on the articular surface as mentioned in a former TiN retrieval study [12].

A coating breakthrough was observed in the patellofemoral joint region of three retrievals. In two cases, the adverse direct contact between the patellar metallic base plate and the metallic femoral component led to abrasive wear, resulting from a patellar maltracking and a patellar dislocation. The restoration of the patellofemoral joint has often been reported as a clinical problem after TKA with instability rates in the range of 1–12% [9, 18]. However, besides pain, dislocation and revision surgery, incorrect loading of the TKA is known to cause patella maltracking.

In the third case of a coating breakthrough, the surgery report showed that revision was necessitated by ligament instability. Moreover, no patella resurfacing was used in combination with the examined TiN-coated mobile-bearing TKA. Therefore, it can be assumed that the native patella eventually led to the coating breakthrough. Two factors might have played an important role within this failure mode: first, the given geometrical nonconformity between the native patella and the femoral component right after primary surgery. This nonconformity might have prevented the retropatellar bone surface from achieving even contact with the coated femoral component. Moreover, the articulation in the beginning of the running-in phase took place only at separated points or along a line, respectively. As a result, the contact surface of the retropatellar joint was reduced. Second, the retropatellar pressure increased significantly after TKA compared to a natural knee [25]. This might have led to high-level contact stresses in combination with the reduced contact surface. Both factors affected wear of the retropatellar bone until the geometrical conformity to the femoral component was achieved. Up to this point, the coating was heavily stressed, considering the applied local stress and the generated third bodies, resulting in a coating abrasion process.

It should be mentioned that the analysis of the clinical data and the evaluation of the corresponding retrievals have demonstrated that the reasons for revision cannot be attributed to the coating breakthroughs. Previous TiN studies with implants made of titanium alloy have suggested that a coating breakthrough was associated with the uncovered substrate material being prone to third-body wear [12, 22]. However, the vulnerability of the CoCrMo TKAs observed within our study was reduced by the increased strength of the substrate material compared to titanium alloys commonly used in total hip arthroplasty. Nonetheless, the barrier for the metal ion release is damaged by a coating breakthrough. Objectively, comparing the total area of the intact TiN surface to the area of coating breakthrough, only a slightly increased ion release could be expected.

### Roughness

Despite a longer implantation period [3], the arithmetical mean surface roughness of the retrievals coated with TiN was comparable with retrieval surfaces made of OxZr or CoCr. Based on the initial mean surface roughness, the absolute roughness levels during in vivo service increased to the range of 0.01 µm for TiN (30.7 months) and doubled to 0.02 µm for uncoated CoCrMo (23.1 months). Thus, the surface roughness remained smoother with the TiN coating, which could be attributed to a reduced polyethylene wear rate, as demonstrated in different knee simulator tests [20, 21].

The absolute values for the maximum profile peak were higher with TiN in contrast to CoCr or OxZr due to the coating technology (e.g. deposition of titanium droplets on the surface). Nevertheless, the decrease in the peak height for TiN and OxZr as a result of in vivo loading could be attributed to a smoothening effect which occurred during the running-in phase. An increase in the maximum peak height, as seen with CoCr retrievals [3], might indicate the poor scratch resistance of CoCr in contrast to TiN and OxZr.

Finally, the condyles of the retrievals were found to be slightly rougher on the medial side than lateral. This was consistent with former retrieval studies of Heyse et al. [13] and Brandt et al. [3] and could be attributed to the primarily medial applied contact forces, as mentioned by Halder et al. [11]. However, this behaviour was already observed in our control group with new, unused surfaces and indicated a difference as a result of the polishing process.

### Coating thickness

According to Lützner et al. [16], TKAs are subjected to a number of motion cycles that amount to approximately 6.500 steps taken daily. During a combination of the sliding and rollback knee movement, wear occurs at the counterfaces of the bearing. Our study demonstrated low abrasive wear of the TiN coating even after several years of in vivo loading. All measured thickness values were found to be within the initial coating thickness range. Unfortunately, this study was not able to make a general statement about the coating thickness decrease rate per year because on the one hand, the initial coating thickness of new femoral components that had never been implanted was not measured due to the destructive measurement method. On the other hand, wear to the counterface is always dependent on specific patient-related factors such as implant position or level of activity [30]. Further, it should be noted that the initial coating thickness is not a general defined value. Rather, the initial coating thickness is batch related (e.g. depends on the number of implants in the coating chamber) and is therefore defined as a range. However, the coating thickness decrease rate per year was minimal, which was confirmed by the fact that the measured thickness values did not deteriorate in line with the implantation period.

A conclusion was not reached about the most stressed side due to the minimal deviations in coating thickness between the medial and lateral condyles. The smallest coating thickness was measured on the femoral component that was revised due to a patella dislocation after it was used for more than 5 years. The corresponding retrieved patella base plate showed patterns of a metal-on-metal contact with the femoral component. Thus, a severe third-body wear scenario on the counterfaces could be expected, representing a worst-case scenario in TKA.

The present study dealt with some limitations that should be explained as follows: all evaluated parameters were based on prematurely revised implants. Thus, the results of this study did not exactly represent the in vivo performance of TiN-coated TKAs without any complications. Another limitation was the limited number of retrievals, which was attributed to the limited number for this type of TKA in the retrieval archive of the hospital.

In addition, uncoated, retrieved femoral components of identical design would have presented the ideal group when comparing the surface characteristics and abrasive wear of CoCrMo substrates with those of coated, retrieved components. However, this reference group was not available. Further, the level of patient activity as well as the reasons for revision surgery was not considered within the analysis. Nonetheless, the 25 analysed retrieved femoral components gave an exclusive insight into the midterm wear resistance of TiN coatings in TKA. Finally, as radiological images were not available, malalignments, particularly in the patella, could not be proven in all cases.

## Conclusion

The semi-quantitative assessment method used represents a suitable and objective method to evaluate, for the first time, the damage to retrieved TiN-coated TKAs. The low damage scores demonstrated that the TiN coating revealed a high level of wear resistance to the in vivo loading, especially in view of the clinical relevance of third-body particles. This was additionally confirmed by the measured coating thickness, which did not deteriorate in line with the implantation period. Nevertheless, a slight roughening effect of the TiN surface as a result of the in vivo loading was observed on the retrievals in comparison with new non-used femoral components.

Critical coating defects like delamination or coating breakthrough were rare. However, this research proved that the reasons for revision could not be attributed to the damage of the coating. Moreover, these defects were a result of massive plastic substrate deformation or patella maltracking. Therefore, in terms of TiN-coated TKAs, particular attention should be paid post-operatively to a correct patellar tracking in order to avoid wear propagation at the knee arthroplasty component.
